# Fowl Adenovirus 4 (FAdV-4)-Based Infectious Clone for Vaccine Vector Development and Viral Gene Function Studies

**DOI:** 10.3390/v10020097

**Published:** 2018-02-24

**Authors:** Yanlong Pei, Juan C. Corredor, Bryan D. Griffin, Peter J. Krell, Éva Nagy

**Affiliations:** 1 Department of Pathobiology, Ontario Veterinary College, University of Guelph, Guelph, ON N1G 2W1, Canada; ypei@uoguelph.ca (Y.P.); corredor@uoguelph.ca (J.C.C.); bdjgriffin@gmail.com (B.D.G.); 2 Department of Molecular and Cellular Biology, University of Guelph, Guelph, ON N1G 2W1, Canada; pkrell@uoguelph.ca

**Keywords:** fowl adenovirus 4 ON1, infectious clone, ORF16 and ORF17, enhanced green fluorescence protein, recombinant vaccine vector, FAdmid

## Abstract

Fowl adenovirus 4 (FAdV-4) is associated with economically important poultry diseases. Recent studies of fully sequenced genomes of FAdV-4 isolates suggest potential genomic regions associated with virulence and amenable for manipulation and vector development. Direct manipulation of viral genomes is cumbersome, as opposed to that of infectious clones—viral genomes cloned into plasmid or cosmid vectors. In this work, we generated an infectious clone, pFAdV-4 ON1, containing the entire viral genome of a nonpathogenic FAdV-4 (ON1 isolate). pFAdV-4 ON1 was used for targeted deletion of open reading frames (ORFs) 16 and 17 and replacement with the enhanced green fluorescence protein (EGFP) expression cassette to generate recombinant viruses. These viruses were viable, and EGFP was expressed in infected cells. Their replication, however, was significantly reduced with respect to that of the wild-type virus. These observations suggest the potential utility of FAdV-4 as a vaccine vector and the importance of ORFs 16 and 17 for virus replication at wild-type levels. To our knowledge, this is the first report of an infectious clone based on the FAdV-4 genome, and our results demonstrate its utility for studies of virulence determinants and as a platform for either vaccine or gene delivery vectors.

## 1. Introduction

Fowl adenovirus 4 (FAdV-4) is a member of the species *Fowl aviadenovirus C* belonging to the genus *Aviadenovirus*, family Adenoviridae. FAdV-4 is associated with economically important poultry diseases such as inclusion body hepatitis (IBH) and hydropericardium syndrome (HPS) [1,2,3]. The virus infects the liver and kidney and causes an accumulation of a straw-colored fluid in the pericardial sac and enlarged pale and friable liver and kidney [4,5]. The virulence determinants of FAdV-4 and the molecular basis of IBH and HPS pathogenesis are unknown. Previous studies suggest the fiber protein and a 12 kb region containing open reading frames (ORFs) 19, 27, 29, and tandem repeat region E (TR-E) as potential virulence determinants for FAdV-8 and FAdV-4, respectively [3,6,7].

The complete nucleotide sequence has been determined for the genomes of 24 FAdV-4 isolates with nucleotide sequence homologies of 97–99% (see accession numbers GU188428; HE608152; KP295475; KU342001; KU991797; KU587519; KM096544; KX538980; KU558762; KX061750; KU569295; KX090424; KY436520; KU569296; KY436522; KU558761; KY379035; KU558760; KY436521; KY436519; KU245540; KX421403; KX421404; KX421401). One of these isolates is the nonpathogenic FAdV-4 ON1, which was isolated from a Canadian broiler breeder flock with no clinical signs of IBH/HPS [8]. Via experimental infection of specific pathogen-free chickens, we demonstrated that this virus did not cause any clinical signs with evidence of virus replication, tissue distribution, and virus shedding [9]. We determined the complete nucleotide sequence of FAdV-4 ON1 (accession no. GU188428) [8] and showed its ability to induce neutralizing antibodies as well as the expression of genes encoding cytokines, such as interferon gamma (IFNγ) and interleukin 10 (IL-10) in the liver [9]. Therefore, FAdV-4 ON1 is attractive as a vaccine for IBH/HPS and as a virus vector. The left and central regions of the sequenced genomes are conserved, while intertypic variations mainly occur at the right ends. Such variations are thought to be associated with virus virulence [3,10,11,12,13]. Therefore, systematic manipulations of the viral genome can be anticipated to provide insights into virulence determinants and help identify dispensable regions for the development of FAdV 4-based vectors.

Fowl adenoviruses can be engineered by direct manipulation of the viral genomes in vitro [6,14,15] or through the use of infectious clones—the adenoviral genome in FAdmids, which are infectious plasmid or cosmid vectors—generated by homologous recombination in *Escherichia coli* [16,17,18]. Direct manipulation of the viral genome is cumbersome and time-consuming, whilst infectious clones can be easily manipulated and prepared at a large scale. The viral genome is released from the cosmid vector as a linear genome by restriction enzyme digestion and transfected into susceptible cells to generate viable viruses. Fowl adenovirus infectious clones, such as those of FAdV-1 (chicken embryo lethal orphan, CELO, strain) and FAdV-9 (A-2A), have already been useful for studies on virus–host interactions, viral gene function, and identification of nonessential regions for virus engineering [16,17,18,19,20,21]. This study extends the applicability of infectious clones to FAdV-4.

Homologues to FAdV-4 ORF17 are present in virus members of species *Fowl aviadenovirus A* to *E* and *Turkey adenovirus B* and *D*. Fowl adenovirus 4 ORF16 homologues are found in the species *Fowl aviadenovirus A* (FAdV-1), *Fowl aviadenovirus C* (FAdV-4 and -10), *Turkey aviadenovirus D* (Turkey adenovirus-5), and *Goose aviadenovirus A* (Goose adenovirus-4) [8,22,23,24]. However, studies on the importance and function of these ORFs on virus replication are very limited. Homologues to ORF16 are putative ADP-ribosyltransferase family proteins with unknown function [22,25]. Previous studies have shown that FAdV-1 ORFs 16 and 17 are nonessential for virus replication in LMH cells [18]. Recently, we have also shown that ORF17 is not essential for FAdV-9 replication in vitro, though it seems required for replication at wild-type levels [26]. Therefore, we chose ORFs 16 and 17 for targeted deletion to investigate their effects on virus replication and as a foreign gene insertion site. To perform molecular studies on FAdV-4, we first generated an infectious FAdmid clone from the nonpathogenic FAdV-4 ON1, named pFAdV-4 ON1. pFAdV-4 ON1 was used as a template for targeted deletion of ORFs 16 and 17 and replacement with either chloramphenicol acetyl transferase (CAT) or the enhanced-green fluorescence protein (EGFP) expression cassette. The infectious clone pFAdV-4 ON1 (parental clone) and the recombinants pFAdV-4 ON1ΔORF16/17-CAT, pFAdV-4 ON1ΔORF16/17-EGFP-L (EGFP in a leftward orientation), and pFAdV-4 ON1ΔORF16/17-EGFP-R (EGFP in a rightward orientation) generated viable viruses. However, the recombinant viruses replicated at lower titers relative to those of the wild type (FAdV-4 ON1). Therefore, our results suggest that the region containing ORFs 16 and 17, while dispensable, is required for replication at wild-type levels and demonstrate the utility of FAdV-4 ON1 as a platform for the development of vaccine and gene delivery vectors.

## 2. Materials and Methods

### 2.1. Cells and Viruses

The FAdV-4 ON1 strain was isolated from broiler chickens with no clinical signs of IBH or HPS [8]. The virus was propagated in a chicken hepatoma cell line (CH-SAH) as described in [27].

### 2.2. Polymerase Chain Reaction Amplification

PCR reactions were carried out in a 50 μL final volume that included 1 × PCR buffer (200 mM Tris–HCl, 500 mM KCl pH 8.8), 2 mM MgSO_4_, 1 mM dNTPs, 20 pmol of each primer (Table 1), 2 U KOD polymerase, and 100 ng DNA. The PCR conditions were as follows: initial denaturation at 95 °C for 2 min, 35 cycles and a 10 min final extension at 72 °C. Each cycle consisted of denaturation at 95 °C for 15 s, annealing at 52–56 °C for 20 s, and extension at 70 °C for 25 s/kb. The PCR products were purified using a PCR gel purification kit (BioBasic, Markham, ON, Canada).

### 2.3. Construction of FAdV-4 ON1 Infectious Clone

**Figure 1:**
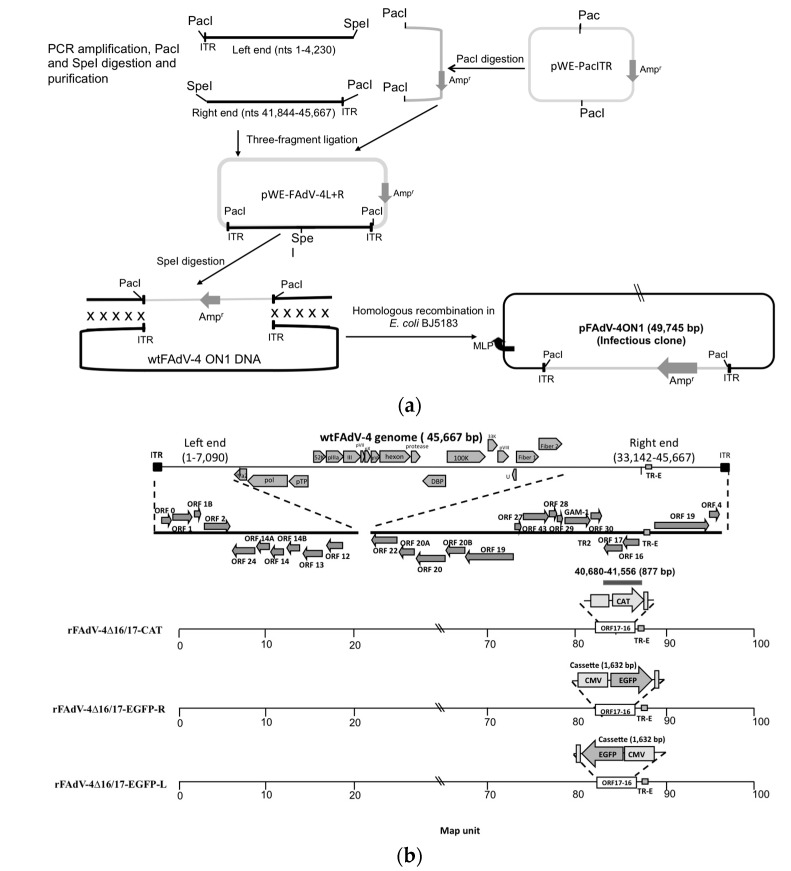

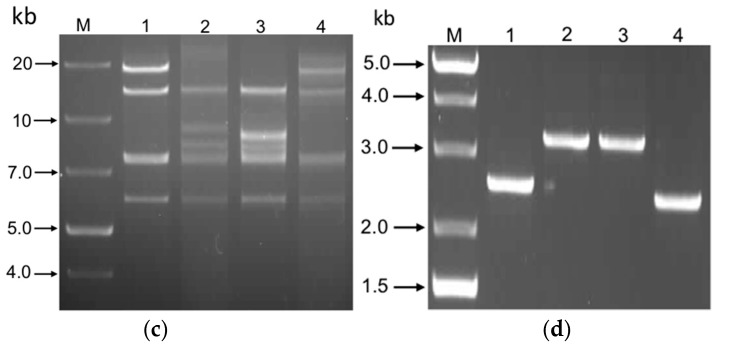
Construction of fowl adenovirus 4 ON1 (FAdV-4 ON1) infectious clone by homologous recombination and generation of ORF16–17 deleted mutant/recombinant viruses. (**a**) The left- and right-end termini (1–4230 and 41,844–45,667, respectively) were PCR-amplified with specific primers (Table 1) to introduce *Pac*I and *Spe*I sites. Polymerase chain reaction (PCR) products were digested with *Pac*I and *Spe*I and purified as described in materials and methods. The modified pWE-15 cosmid was digested with *Pac*I and purified. The digested PCR fragments and pWE-15 were ligated to generate pWE-FAdV-4L+R, the intermediate construct. pWE-FAdV-4L+R was digested with *Spe*I, purified, and co-transformed with the FAdV-4 genome in *E. coli* BJ5183 to generate pFAdV-4 ON1. Viable virus was generated upon transfection of CH-SAH cells with *Pac*I-digested pFAdV-4 ON1 (not shown); (**b**) chloramphenicol acetyl transferase (CAT)-mediated deletion of ORFs 16 and 17 was carried out using the lambda Red recombinase approach as described previously [21,29] to generate pFAdV-4Δ16/17-CAT. Chloramphenicol acetyl transferase was removed by *Swa*I digestion and replaced with the enhanced-green fluorescence protein (EGFP) expression cassette (cytomegalovirus promoter-EGFP coding region-Poly A signal) in both leftward and rightward orientations; (**c**) Infectious clones were verified by *Hind*III digestion: lane M, 1 kb DNA ladder; lane 1, pFAdV-4Δ16/17-CAT (fragment sizes are 16.3 kb, 12.4 kb, 7.7 kb, 7.4 kb and 5.9 kb); lane 2, pFAdV-4Δ16/17-EGFP-R (fragment sizes are 12.4 kb, 9.4 kb, 7.7 kb, 7.4 kb, 5.9 kb and 5.7 kb); lane 3, pFAdV-4Δ16/17-EGFP-L (fragment sizes are 12.3 kb, 9.0 kb, 7.7 kb, 7.4 kb, 6.0 kb and 5.9 kb); lane 4, parental pFAdV-4 ON1 (fragment sizes are 16.4 kb, 12.4 kb, 7.7 kb, 7.4 kb and 5.9 kb); (**d**) viable viruses were generated upon transfection of *Pac*I-digested infectious clones (wild type and recombinants). The stability of the transgenes (*CAT* and *EGFP*) in viable virus genomes was verified after three passages by PCR with specific primers flanking the deleted region; lane M, 1 kb DNA ladder; lane 1, FAdV-4Δ16/17-CAT (2,628 bp); lane 2, FAdV-4Δ16/17-EGFP-R (3,210 bp); lane 3, FAdV-4Δ16/17-EGFP-L (3,210 bp); and lane 4, wtFAdV-4 ON1 (2,453 bp). ITR: inverted terminal repeat.

Molecular cloning techniques and extraction of the viral DNA were performed as described in [17,28,29]. Briefly, for large-scale preparation of viral DNA, CH-SAH cells in 150 mm cell culture dishes were infected with FAdV-4 ON1 (multiplicity of infection, MOI, of 1) and incubated at 37 °C (5% CO_2_) until complete cytopathic effects (CPE) were apparent. After three freeze–thaw cycles, cells and virus were harvested and centrifuged at 3300× *g* for 15 min at 4 °C. The supernatants were ultracentrifuged (100,000× *g*) in 30% sucrose in TNE buffer (10 mM Tris–HCl pH 7.5, 100 mM NaCl, 1 mM EDTA pH 8.0) at 4 °C for 2 h. The viral pellets were resuspended in TNE buffer followed by proteinase K treatment, DNA extraction with phenol and chloroform, and ethanol precipitation. The left-end fragment (nucleotides (nts) 1–4267) was PCR-amplified with primer pair FAdV4R-End*Pac*I-F and FAdV4R-End*Spe*I-R, and the right-end fragment (nts 41,222–45,663) was amplified with primer pair FAdV4L-End*Spe*I-F and FAdV4L-End*Pac*I-R (Table 1). The modified pWE-15 cosmid was digested with *Pac*I, and the PCR products were digested with both *Pac*I and *Spe*I. After gel purification (QIAquick II Gel Extraction Kit, Qiagen, Mississauga, ON, Canada), the three-fragment ligation (left- and right-end fragments and pWE-15) was carried out as described in [17] to generate the pWE-FAdV-4L+R intermediate construct, which were confirmed by sequencing. After digestion with *Spe*I and gel purification, 1 μg of linearized pWE-FAdV-4L+R and 3 μg of FAdV-4 ON1 DNA were co-transformed into *E. coli* BJ5183. Infectious clone pFAdV-4 ON1 generated upon homologous recombination (Figure 1a) was verified by *Hind*III digestion.

### 2.4. Construction of ORFs 16- and 17-Deleted Mutant/Recombinant Viruses

The generation of ORF16–17-deleted infectious clones carrying foreign genes is depicted in Figure 1a,b. All manipulations were carried out as described in [21,29]. A fragment containing ORFs 16 and 17 was deleted and replaced with the chloramphenicol acetyl transferase (*CAT*) gene using the λ Red recombinase system. Bacterial clones were selected with chloramphenicol. Briefly, *E. coli* DH10B carrying pFAdV-4 ON1 was transformed with pJW103 by electroporation. pJW103 carries the λ Red recombinase and kanamycin resistance genes. The λ Red recombinase gene is under the control of the arabinose inducible promoter [30]. The *CAT* gene cassette was PCR-amplified from pKD3 with the primer pair FAdV-4ORF17CAT-F and FAdV-4ORF16CAT-R which contains homologous arms upstream and downstream of the fragment containing ORFs 16 and 17 (each one of 52 bp), *Swa*I sites (ATTTAAAT), and *CAT* gene cassette specific sequences (Table 1). The PCR products were gel purified and electroporated into *E. coli* DH10B carrying both pFAdV-4 ON1 and pJW103. Arabinose was added to a final concentration of 0.1%, and the cultures were incubated at 30 °C overnight. The bacteria were plated onto Luria-Bertani (LB)-agar supplemented with 100 μg/mL ampicillin and 34 μg/mL chloramphenicol. The *CAT*-marked infectious clone with a deletion of ORFs 16 and 17 (pFAdV4Δ16/17-CAT) was verified by *Hind*III digestion. The enhanced-green fluorescent protein (*EGFP*) gene cassette (cytomegalovirus, CMV, promoter-EGFP coding region-poly A) was amplified from the pEGFP-N1 vector with primer pair EGFPca*Swa*I-F and EGFPca*Swa*I-R (which both carry the *Swa*I sites), followed by gel-purification and digestion with *Swa*I, which generates blunt-ended DNA fragments. Blunt-end ligation of *Swa*I-digested DNA fragments, EGFP cassette, and pFAdV4Δ16/17-CAT was carried out with T4 DNA ligase (Invitrogen, Burlington, ON, Canada) at 14 °C for at least l6 h. The *Pac*I-digested clones, pFAdV4Δ16/17-CAT, pFAdV-4Δ16/17-EGFP-R (EGFP in rightward orientation), and pFAdV-4Δ16/17-EGFP-L (EGFP in leftward orientation) were transfected into CH-SAH cells by Lipofectamine 2000 (ThermoFhisher Scientific, Mississauga, ON, Canada) to generate the recombinant viruses rFAdV-4Δ16/17-CAT, rFAdV-4Δ16/17-EGFP-R, rFAdV-4Δ16/17-EGFP-L, respectively. The rescued virus (resFAdV-4Δ16/17) was generated by deletion of the CAT gene from pFAdV4Δ16/17-CAT and replacement with a fragment containing ORFs 16 and 17. The infectious clones were verified by *Hind*III digestion of viral DNA (Figure 1c), and the stability of the recombinant regions over three passages was verified by PCR (Figure 1d) with primers flanking the deleted region, named FAdV-4Ver-F and FAdV-4Ver-R (Table 1).

### 2.5. One-Step Growth Curves

The virus growth kinetics were determined in CH-SAH cells as described in [25]. Briefly, cells were infected with rFAdV-4Δ16/17-EGFP-R, rFAdV-4Δ16/17-EGFP-L, and wtFAdV-4 ON1 (MOI of 5), and the virus was harvested at the indicated times post-infection (p.i.). Plaque assays were carried out as described in [27].

## 3. Results

### 3.1. Generation of FAdV-4 Infectious Clone (pFAdV-4 ON1) and ORF16–17-Deleted Mutant/Recombinant Viruses

We first generated the intermediate plasmid construct pFAdV-4R+L containing both genomic termini (Figure 1a). The left-end fragment is comprised of nts 1–4230, whilst the right-end fragment consists of nts 41,844–45,667. Both termini were PCR-amplified with specific primers to incorporate *Pac*I and *Spe*I restriction sites. *Pac*I sites allowed the cloning of both termini into the modified pWE-15 cosmid vector, while both termini were ligated into the *Spe*I site. Upon digestion with *Spe*I, the linearized pFAdV-4R+L was co-transformed with FAdV-4 ON1 genomic DNA into *E. coli* BJ5183 to generate pFAdV-4 ON1 infectious clone by homologous recombination. pFAdV-4 ON1 was subsequently linearized with *Pac*I and transfected into CH-SAH cells. Cytopathic effects appeared at 5 days post-transfection suggesting that *Pac*I-digested pFAdV-4 ON1 gives rise to viable virus.

It was possible to engineer FAdV members of *Fowl aviadenovirus species A* (FAdV-1), *D* (FAdV-9), and *E* (FAdV-8) as recombinant poultry vaccines and gene delivery systems [14,15,17,19,21,31,32,33] because of the presence of dispensable regions in their genomes. Therefore, we reasoned that FAdV-4, which belongs to the species *Fowl aviadenovirus C*, would also contain dispensable regions for virus engineering. Open reading frames 16 and 17 have been shown to be dispensable for in vitro replication of FAdV-1 and -9 [18,26]. We therefore reasoned that these ORF homologues were also dispensable for FAdV-4 replication and suitable for foreign gene cloning/replacement. Open reading frames 16 and 17 were deleted and replaced with a CAT cassette (Figure 1b) by targeted homologous recombination in pFAdV-4 ON1 using the lambda Red recombinase system as described in [21,29]. Transfection of CH-SAH cells with *Pac*I-digested pFAdV-4Δ16/17-CAT resulted in viable virus (rFAdV-4Δ16/17-CAT) suggesting that ORFs 16 and 17 are dispensable for virus replication. To determine the utility of FAdV-4 ON1 as a vector for vaccine or gene delivery, the *CAT* gene was removed from pFAdV-4Δ16/17-CAT with *Swa*I and replaced with the *Swa*I-digested EGFP expression cassette. Since *Swa*I generates blunt-ended DNA fragments, it was possible to clone the EGFP cassette in both orientations. *Hind*III banding patterns and direct sequencing confirmed the orientation of EGFP and the identity of the infectious clones, wild type and recombinants (Figure 1c). The resulting recombinant infectious clones, pFAdV-4Δ16/17-EGFP-R (EGFP cassette in rightward orientation) and pFAdV-4Δ16/17-EGFP-L (EGFP cassette in leftward orientation), were digested with *Pac*I and transfected into CH-SAH cells to generate viable viruses named rFAdV-4Δ16/17-EGFP-R and rFAdV-4Δ16/17-EGFP-L. PCR amplification with specific primers flanking the deletion/insertion region (Table 1) confirmed virus stability after four passages (Figure 1d).

### 3.2. Viral Growth Kinetics

**Figure 2:**
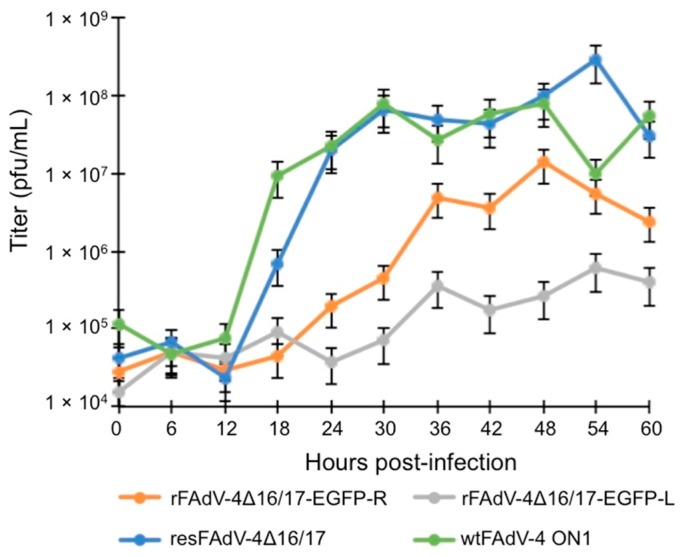
One-step growth curves. Chicken hepatoma cells (CH-SAH) were infected with wild-type (wt), rescued, and recombinant viruses (multiplicity of infection of 5), and virus was harvested at the indicated time points and titrated as described in [27]. Total virus titers were determined in two technical repeats by plaque assay and expressed as plaque-forming units (pfu)/mL.

The plaque morphology among the different recombinant viruses was similar (not shown). However, growth kinetics differed between wild-type and recombinant viruses (Figure 2). Rescued virus, resFAdV-4 Δ16/17, was generated to rule out any effects of secondary mutations resulting from manipulation, and no differences were noted between the two growth curves. Relative to wild-type and resFAdV-4 Δ16/17 viruses, rFAdV-4Δ16/17-EGFP-R and rFAdV-4Δ16/17-EGFP-L replicated nearly 3 and 2 logs slower, respectively, suggesting that the fragment containing ORFs 16 and 17, while not essential, is required for virus replication at wild-type levels. rFAdV-4Δ16/17-EGFP-R replicated 1 log faster than rFAdV-4Δ16/17-EGFP-L (Figure 2) suggesting the importance of foreign gene orientation within this region on virus replication.

### 3.3. Transgene Expression Upon Infection with Recombinant Viruses

**Figure 3:**
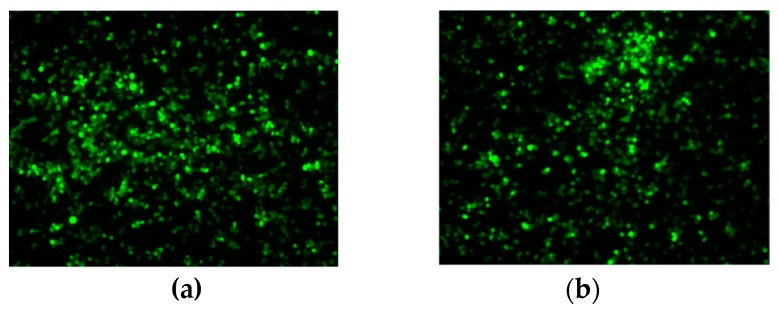
Enhanced-green fluorescence protein (EGFP) expression in CH-SAH infected with recombinant FAdV-4 viruses. CH-SAH cells were infected with (**a**) rFAdV-4ON1∆16-17EGFPCA-R and (**b**) rFAdV-4ON1∆16-17EGFPCA-L and examined by fluorescence microscopy. Images were taken three days post-infection. Magnification 50×.

Cells infected with either rFAdV-4Δ16/17-EGFP-R or rFAdV-4Δ16/17-EGFP-L expressed EGFP (Figure 3) at seemingly comparable intensities per cell. These observations show that the region containing ORFs 16 and 17 is dispensable for virus replication in vitro and is suitable as an insertion site for expressible foreign genes.

## 4. Discussion

FAdV-4 is the etiological agent for IBH and HPS, which are economically important diseases in the poultry industry. Prevention of IBH and HPS currently involves the use of inactivated or live attenuated viruses and subunit vaccines [34,35,36,37]. Because of the negative impacts of FAdV-4 on the poultry industry, studies on virus–host interactions and the identification of virulence determinants are paramount for the development of safe and efficient vaccines.

Nucleotide sequence analyses of pathogenic and nonpathogenic FAdV-4 show intertypic variations at the right end of the genome that could be associated with virulence. Relative to the sequenced genomes of nonpathogenic viruses (KR-5 and ON1), such intertypic variations consist of insertions and deletions in ORFs 19, 27, 29, and 19A and the TR-E region between ORFs 16 and 19A [3,10,11,12,13]. Other variations include insertions in the GA repeats between the pX and pVI genes and the TC repeats between the protease and DNA-binding protein genes [10]. In the present study, we have successfully generated the pFAdV-4 ON1 infectious clone that will facilitate the systematic study of these nucleotide sequence variations and their association with virulence, targeted genetic manipulations for the generation of safer live-attenuated FAdV-4 vaccines, and the identification of additional non-essential genomic regions for further development of virus-based vaccine platforms.

In the present study, we showed that ORFs 16 and 17 are required for replication at wild-type levels. Consistent with our recent findings of ORF17 in FAdV-9 [26], viruses lacking the fragment containing ORFs 16 and 17 replicate at lower titers, suggesting a role of these ORFs for virus replication at wild-type levels. While one possibility for the decreased levels of virus replication is that deletion of these ORFs may have had polar effects in the expression of other upstream or downstream ORFs, we do not have a transcriptional map of FAdV-4 to evaluate this possibility. Despite their reduced replication, recombinant viruses expressed a foreign gene (*EGFP*) in infected cells (CH-SAH), suggesting that the region containing ORFs 16 and 17 is suitable as a cloning/replacement site. The orientation of the foreign gene cassette within this genomic region had dramatic effects on virus replication. rFAdV-4Δ16/17-EGFP-R (EGFP cassette with rightward orientation) replicated better (>1 log) than rFAdV-4Δ16/17-EGFP-L (EGFP cassette with leftward orientation). We speculate that the leftward EGFP transcript generated from the CMV promoter may overlap and have antisense effects on viral transcripts of upstream ORFs with a rightward orientation: ORFs 8 (Gam-1), 42, 43, 28, and 29. Indeed, using DNA Poly(A) Signal Miner output software [38], polyadenylation sites for these ORFs are predicted at nts 44,710 and 45,461—which map inside and outside ORF4, respectively—giving rise to large transcripts that can overlap the leftward EGFP transcript, probably causing deregulation of the expression of these ORFs. Gam-1 has been shown to be essential for FAdV-1 replication [39], and decreased levels of this gene by leftward EGFP antisense transcripts may also compromise FAdV-4 replication. The human adenovirus 2 fiber transcript is known to be transcribed to nearly the end of the viral genome [40]. If this is also the case for FAdVs, it is also likely that the leftward EGFP may partially overlap and have antisense effects on the fiber gene transcripts, resulting in decreased expression of fiber genes. EGFP expression levels in cells infected with rFAdV-4Δ16/17-EGFP-L and rFAdV-4Δ16/17-EGFP-R were nearly similar, suggesting that antisense effects of a leftward EGFP transcript on virus transcripts are unlikely. Promoter interference has also been described in virus vectors [41], and thus the CMV promoter in the leftward orientation is likely to have negative effects on the expression of viral genes that would collectively contribute to wild-type levels of replication.

The utility of other FAdVs such as FAdV-1, -8, -9, and -10 as vaccine vectors has been demonstrated [14,15,16,17,31]. Recombinant FAdV-8 and -10 viruses were generated by direct manipulation of the viral genomes [6,14], whilst recombinant FAdV-1 and -9 viruses were generated from infectious FAdmid clones [16,17]. Here, we showed the utility of pFAdV-4 ON1 as an infectious clone for molecular studies and rapid engineering of improved FAdV-4-based vectors for vaccine and gene delivery purposes.

In conclusion, to our knowledge, this work reports the first infectious clone containing the entire FAdV-4 genome. This study is also the first to report the generation of FAdV-4-based recombinant viruses. We demonstrated that ORFs 16 and 17, although dispensable, are required for replication at wild-type levels and amenable to manipulation to generate viable mutant/recombinant viruses. Relative to the nonpathogenic FAdV-4 ON1, nucleotide sequence changes have been identified in some regions of virulent strains of FAdV-4 [3,10,11,12,13]. Therefore, this infectious clone is an excellent tool for the evaluation and confirmation of whether such nucleotide changes could lead to virulence and for further studies on viral gene function and host–virus interactions.

## Figures and Tables

**Table 1:** List of Primers.

Primer	Sequence (5’-3’)	Location	Purpose
FAdV4R-EndPacI-F	*agtc*TTAATTAAcatcatcttatataaccgcgtcttttgacac	1–31	Generate a 4.2-kb fragment (left end genomic region) with PacI and SpeI restriction sites
FAdV4R-EndSpeI–R	*agtc*ACTAGTcttacctcggatgaactatgccactg	4205–4230	
FAdV4L-EndSpeI-F	cacaaggtacatgaatcACTAGTaatggtc	41,844–41,873	Generate a 3.8-kb fragment (right end genomic region) with PacI and SpeI restriction sites
FAdV4L-EndPacI-R	*agtc*TTAATTAAcatcatcttatataAccgcgtcttttgacacacttac	45,631–45,667	
FAdV-4ORF17CAT-F	catgacacagagggaggagactgcgagtaatcacctttaattattaacagctATTTAAAT**gtgtaggctggagctgcttc**	40,629–40,680	Introduce CAT-gene expression cassette with upstream and downstream homologous arms to ORFs17 and 16, respectively
FAdV-4ORF16CAT-R	gagcaggaaaatctgcagagcactcttttggcggtcccgtgtgcggtgggtaATTTAAAT**catatgaatatcctccttagttc**	41,557–41,608	
FAdV-4Ver-F	cgactcctcctctttgtgggc	40,085–40,105	Verification
FAdV-4Ver-R	gcggcatctcctagaatgagg	42,518–42,538	Verification
EGFPcaSwaI-F	*agctgc*ATTTAAATgtattaccgccatgcattag	4717–3	EGFP cassette amplification
EGFPcaSwaI-R	*agctgc*ATTTAAATccacaactagaatgcagtg	1597–1615	

Restriction enzyme sites are capitalized; Sequences in bold are CAT gene cassette specific; Underlined sequences are pN1-EGFP specific. The location is based on pEGFP-N1; Primer location is based on the complete nucleotide sequence of fowl adenovirus 4 ON1 (FAdV-4ON1). Accession GU188428; Italicized sequences are extra nucleotides for restriction enzyme digestion.
